# SRE-FMaps: A Sinkhorn-Regularized Elastic Functional Map Framework for Non-Isometric 3D Shape Matching

**DOI:** 10.3390/jimaging11120452

**Published:** 2025-12-16

**Authors:** Dan Zhang, Yue Zhang, Ning Wang, Dong Zhao

**Affiliations:** 1 School of Computer, Qinghai Normal University, Xining 810016, China; danz@mail.bnu.edu.cn (D.Z.); 13935802804@163.com (Y.Z.); zhaodong@mail.sdu.edu.cn (D.Z.); 2 The State Key Laboratory of Tibetan Intelligence, Xining 810016, China; 3 The State Key Laboratory of Tibetan Intelligent Information Processing and Application, Qinghai Normal University, Hutai, Xining 810008, China; 4 Academy of Plateau Science and Sustainability, People’s Government of Qinghai Province and Beijing Normal University, Xining 810016, China

**Keywords:** functional maps, elastic functional basis, sinkhorn algorithm, cosine distance

## Abstract

Precise 3D shape correspondence is a fundamental prerequisite for critical applications ranging from medical anatomical modeling to visual recognition. However, non-isometric 3D shape matching remains a challenging task due to the limited sensitivity of traditional Laplace–Beltrami (LB) bases to local geometric deformations such as stretching and bending. To address these limitations, this paper proposes a Sinkhorn-Regularized Elastic Functional Map framework (SRE-FMaps) that integrates entropy-regularized optimal transport with an elastic thin-shell energy basis. First, a sparse Sinkhorn transport plan is adopted to initialize a bijective correspondence with linear computational complexity. Then, a non-orthogonal elastic basis, derived from the Hessian of thin-shell deformation energy, is introduced to enhance high-frequency feature perception. Finally, correspondence stability is quantified through a cosine-based elastic distance metric, enabling retrieval and classification. Experiments on the SHREC2015, McGill, and Face datasets demonstrate that SRE-FMaps reduces the correspondence error by a maximum of 32% and achieves an average of 92.3% classification accuracy (with a peak of 94.74% on the Face dataset). Moreover, the framework exhibits superior robustness, yielding a recall of up to 91.67% and an F1-score of 0.94, effectively handling bending, stretching, and folding deformations compared with conventional LB-based functional map pipelines. The proposed framework provides a scalable solution for non-isometric shape correspondence in medical modeling, 3D reconstruction, and visual recognition.

## 1. Introduction

Three-dimensional shape matching is a core problem in computer vision and geometric processing, whose essence is to establish point-wise correspondences between models while preserving geometric or semantic consistency. This task plays a pivotal role across multiple critical application scenarios. In medical imaging, 3D organ modeling enables precise anatomical simulation, supporting drug response assessment and disease mechanism analysis [1]; in face modeling, non-rigid 3D correspondence is fundamental for handling expression variations, directly influencing face recognition robustness [2]; and in industrial inspection, deformation-aware surface recovery ensures accurate structural defect detection in mechanical components, thus safeguarding product reliability [3]. Across these scenarios, non-isometric deformation—characterized by local nonlinear geometric variations such as bending, twisting, and stretching—poses a major challenge, making robust non-isometric matching a prerequisite for real-world deployment.

However, existing 3D matching techniques exhibit critical limitations under such deformation. Traditional Laplace–Beltrami (LB) operator-based methods [4,5] perform reliably in near-isometric conditions due to the isometry-invariant nature of spectral eigenfunctions, which effectively encode global topology. Yet, their low-frequency eigenbases inherently suppress high-frequency signals, rendering them incapable of capturing sharp curvature transitions or localized surface wrinkles. As a result, LB-based pipelines lose discriminative detail under elastic or articulated deformation. To overcome this limitation, our framework replaces LB eigenfunctions with elastic spectral bases [6] that explicitly encode local geometric distortion, thereby recovering deformation-sensitive correspondence that LB operators inherently overlook.

Meanwhile, deep learning-based approaches [7,8] have shown promising matching capabilities but rely heavily on large-scale annotated datasets and supervised training. Their high data dependence and limited generalization to rare or unseen deformation patterns restrict their adoption in industrial and medical environments where labeled data is scarce. Moreover, the high computational burden makes them impractical for large-scale or real-time applications. In contrast, our framework leverages an entropy-regularized Sinkhorn transport module with sparse kernel acceleration to efficiently estimate bijective correspondences without requiring any labeled data, achieving scalability while maintaining robustness.

To address the aforementioned limitations, we propose a Sinkhorn-Regularized Elastic Functional Map framework (SRE-FMaps) for non-isometric 3D shape matching. Unlike the standard elastic basis method [6], which relies on unstable Iterative Closest Point (ICP) [9] refinement, and traditional Sinkhorn approaches [10], which are limited by the Laplace–Beltrami operator, our framework uniquely synergizes these components. SRE-FMaps introduces a non-orthogonal elastic spectral basis derived from the Hessian of thin-shell deformation energy [6], enabling sensitivity to extrinsic deformations and fine-scale curvature variations. In addition, an entropy-regularized Sinkhorn transport module [10] with sparse kernel acceleration is incorporated to compute a bijective and computationally efficient initial correspondence. Intuitively, while the elastic basis acts as a “deformation-aware lens” to capture high-frequency details, the Sinkhorn regularization functions as a “soft assignment filter”, ensuring global consistency without getting trapped in local minima. Furthermore, a multi-scale cosine-based elastic distance metric is proposed to jointly encode global topology and local geometric detail, enabling both matching and classification within a unified framework.

The main contributions of this work are summarized as follows:

- Elastic deformation-aware spectral representation. We propose SRE-FMaps, a Sinkhorn-Regularized Elastic Functional Map framework that replaces conventional LB eigenbases with thin-shell-derived elastic basis functions, greatly enhancing sensitivity to high-frequency non-isometric deformation.
- Scalable entropy-regularized optimal transport correspondence. We integrate entropy-regularized Sinkhorn transport with sparse kernel acceleration to establish bijective and computationally efficient initialization, achieving linear complexity while maintaining high correspondence accuracy.
- Unified matching and classification via elastic cosine distance. We design a multi-scale cosine-based elastic distance metric that fuses global spectral information with local geometric features, enabling reliable evaluation in both 3D correspondence and classification tasks.

The remainder of this paper is organized as follows: Section 2 reviews related work on functional maps and deep shape matching strategies. Section 3 details the proposed SRE-FMaps framework, including the sparse Sinkhorn initialization and elastic basis construction. Section 4 presents the experimental results and performance comparisons on standard datasets. Finally, Section 5 concludes this paper and discusses future directions.

## 2. Related Work

Since 2012, the theory of functional maps [4] and its combination with geometric learning have gradually become the core research framework in the field of 3D non-rigid shape matching. Early work focused on mining the intrinsic geometric properties of shapes (such as Laplace–Beltrami spectral features [5]) and optimizing map constraints (such as orientation preservation and feature alignment), laying the theoretical foundation for low-dimensional matching based on spectral space. In addition, innovative methods based on deep learning continue to emerge, combined with data-driven strategies, to improve the accuracy of shape matching and promote the continued development of this field. This section will introduce traditional functional maps and deep functional maps.

### 2.1. Traditional Functional Maps

Traditional functional maps, first introduced by Ovsjanikov et al. [4], reformulate non-rigid 3D shape matching as a linear mapping problem by enforcing geometric consistency constraints. Correspondence refinement is typically achieved through least-squares optimization combined with Iterative Closest Point (ICP) post-processing [9]. However, such pipelines often suffer from symmetric inversion artifacts in point-to-point recovery.

Subsequent improvements aimed to address this instability. Corman et al. [11] resolved directional ambiguity via supervised descriptor learning and vector field regularization, while Nogneng et al. [12] strengthened local consistency by enforcing operator commutativity constraints. Huang et al. [13] proposed AdjointFmaps, introducing bidirectional coupling through adjoint operators. Poulenard et al. [14] further enhanced robustness by integrating topological invariants as explicit constraints. To improve resolution, Melzi et al. [15] introduced ZoomOut, which progressively upsamples coarse maps via spectral refinement, and Hu et al. [16] incorporated multi-scale spectral manifold wavelets (SMWs) [17] for richer feature encoding.

Despite their success, traditional functional maps fundamentally rely on Laplace–Beltrami (LB) eigenfunctions [5], which primarily capture intrinsic low-frequency structure and are insensitive to extrinsic deformations such as stretching and compression. To overcome this limitation, Magnet et al. [18] proposed ScalableFmaps for dense matching without mesh simplification, while Hartwig et al. [6] introduced an elastic spectral basis derived from thin-shell deformation energy. By encoding high-frequency curvature responses directly into the basis, the latter significantly improves alignment in regions with large geometric distortion.

### 2.2. Deep Functional Maps

Existing methods built on the functional map framework predict functional maps by learning feature representations robust to discretization variations, thereby advancing the development of supervised and unsupervised learning paradigms in 3D shape analysis. The deep functional map framework (FMNet), proposed by Litany et al. [7], models shape matching as a supervised correspondence problem—one that learns functional relationships between shapes via neural networks to achieve more flexible global matching than traditional point-to-point map approaches. Inspired by FMNet, subsequent studies have widely embraced functional maps (FMs) as core matching modules. However, conventional approaches rely heavily on approximate isometric assumptions and necessitate elaborate post-processing pipelines—this fundamentally restricts their applicability to scenarios involving non-isometric deformations.

Subsequent studies inspired by FMNet mostly use FMs as the matching layer, but traditional methods rely on approximate isometry assumptions and have complex post-processing, making them difficult to adapt to non-isometric deformation scenarios. The deep geometric matching framework that has emerged in recent years has broken through this limitation through multidimensional innovations. The Deep Shells framework by Eisenberger et al. [19] incorporates Smooth Shells into an end-to-end architecture, processing both intrinsic and extrinsic geometric information via entropy-regularized optimal transport and spectral CNN features [20]. Building on DiffusionNet [8], Donati et al. [21] developed DUO-FMNet using complex-valued functional maps [22] to achieve directional awareness, enabling unsupervised learning of orientation-sensitive features. Hu et al. [23] proposed RFMNet, synergizing diffusion-based features with optimal transport theory to reduce dependency on handcrafted descriptors while enhancing geometric fusion. Magnet et al. [18] further advanced efficiency with DiffZO, enabling large-scale correspondence through differentiable Zoomout optimization and topology-aware constraints.

The most recent advances have focused on addressing computational scalability and unsupervised robustness. For instance, Magnet et al. [24] proposed a memory-scalable learning pipeline to handle dense meshes, while others have integrated synchronous diffusion models to ensure smooth correspondence without supervision [25]. Furthermore, deep frequency-aware networks have been introduced to enhance spectral stability [26]. Despite these significant strides, ensuring bijective correspondence efficiently without heavy data dependence remains a challenge, which our SRE-FMaps aims to address.

In summary, the review of the existing literature reveals a critical dichotomy in non-isometric shape matching. While traditional spectral methods are mathematically elegant and efficient, their reliance on the isometry-invariant Laplace–Beltrami operator fundamentally limits their ability to capture high-frequency deformations like stretching and bending. Conversely, although deep learning approaches offer improved flexibility, they suffer from heavy dependence on large-scale annotated data and lack generalization to unseen deformation types. These observations suggest a clear need for a framework that incorporates the deformation sensitivity of physical models (such as elastic thin-shell energy) with the computational efficiency of modern optimization techniques, without the constraints of supervised training. This motivates the design of our SRE-FMaps, which bridges this gap by regularizing elastic functional maps with the Sinkhorn algorithm.

## 3. Methods

### 3.1. Overview of the SRE-FMaps Framework

**Figure 1:**
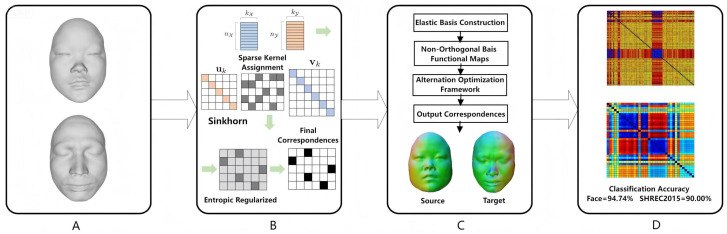
The framework of SRE-FMaps for non-isometric 3D shape matching in imaging applications. (**A**) Input 3D Models; (**B**) Initial Map Calculation; (**C**) Elastic Basis Calculation; (**D**) Distance Calculation and Classification.

Figure 1 illustrates the framework of the non-isometric 3D shape matching algorithm based on elastic functional maps. The specific procedural steps are as follows: (A). Input 3D Models: The input 3D models undergo non-isometric transformations, where their geometries are deformed in a manner that does not preserve the original distance and angle relationships. This deformation causes stretching, compression, bending, or twisting in specific regions, thereby altering local or global geometric properties. (B). Initial Map Calculation: Sparse kernel allocation is performed on the input models. An entropy-regularized transport plan is generated through Sinkhorn iterations, from which the final correspondence is extracted to complete the initial map computation. (C). Elastic Basis Calculation: The SRE-FMaps framework is constructed to generate a non-orthogonal basis functional map. An alternating optimization framework is employed to refine the mapping, ultimately outputting the correspondence between the two models. (D). Distance Calculation and Classification: Based on the established correspondence, the distance between models is computed, followed by classification to evaluate and output the classification accuracy.

**Figure 2:**
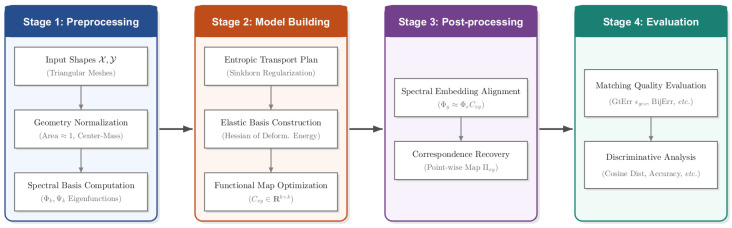
The methodological pipeline of the proposed study. It consists of four main stages: Pre-processing (basis construction), Model Building (SRE-FMaps core algorithm), Post-processing (correspondence recovery), and Evaluation (performance assessment).

To strictly evaluate the proposed method within a comprehensive experimental context, we adopt a systematic methodological pipeline, as illustrated in Figure 2. This pipeline encompasses four sequential stages: Stage 1 (Pre-processing) performs data standardization and spectral basis construction to establish the geometric representation space; Stage 2 (Model Building) implements the core SRE-FMaps algorithm, integrating Sinkhorn initialization and elastic functional map optimization; Stage 3 (Post-processing) converts the computed functional maps into point-to-point correspondences; and Stage 4 (Evaluation) quantifies performance using matching metrics (e.g., geodesic error, bijectivity error, etc.) and classification metrics (e.g., cosine distance [27], etc.).

### 3.2. Initial Map Calculation

The initial point-by-point correspondence is computed through the following procedure.

First, we establish the link between the functional representation and spatial mapping. By leveraging the property that the functional map aligns the spectral embeddings of two shapes, the point-to-point map $T_{xy}$ can be recovered via a nearest neighbor search in the spectral domain. Detailed mathematical derivations regarding the adjoint operators and the consistency between spectral and spatial representations are provided in Appendix A to maintain focus on the core framework.

Next, the idea of extracting point-by-point map $T_{xy}$ by traversing all points on the shape is realized:

(1) $$T_{xy}=\mathrm{NNsearch}\left(\Phi_{y},\Phi_{x}D_{xy}^{T}\right)$$

where NNsearch(p,q) returns the point in *p* closest to query *q*. This step effectively converts the spectral alignment into spatial probability distributions.

Then, the spectral embedding alignment problem is treated as a linear assignment problem to be solved, where the point-to-point map Π is a doubly stochastic matrix and its search space is Q. The problem can be formulated as follows:

(2) $$P_{xy}=\underset{\Pi \in Q}{\arg\min}{\langle d,\Pi \rangle }_{F}$$

where $Q=\{\Pi |\Pi \in R^{n\times n},\;\Pi 1_{n}=\mu_{x},\;\Pi^{T}1_{n}=\mu_{y}\}$, $\mu_{x}$ and $\mu_{y}$ are predefined initial masses for each vertex of *x* and *y*, respectively, and $d\in R^{n\times n}$ is the pairwise Euclidean distance matrix between aligned embeddings: $d_{ij}={∥\Phi_{x}\left(i,:\right)D_{xy}-\Phi_{y}\left(j,:\right)∥}^{2}$.

Finally, the Sinkhorn algorithm is adopted to solve the linear assignment problem and construct a sparse kernel assignment matrix. This matrix only retains the distance values of a few nearest neighbors for each point (denoted as N(i) for the nearest neighbor set of the *i*-th point on *X*), which is expressed as follows:

(3) $$K^{\lambda }={\left[k_{ij}^{\lambda }\right]}_{i=1,2,\ldots ,n_{x},j\in N\left(i\right)}$$

The sparse kernel generated by Sinkhorn iteration improves the accuracy and bijectivity of the initial correspondence without significantly increasing the runtime, and the final initial point-wise correspondence is derived from this iteration.

### 3.3. SRE-FMaps

Based on the obtained initial map, solving for the elastic basis requires constructing the core operators of the functional map method adapted to non-orthogonal basis functions. Unlike standard spectral bases, the elastic basis functions are not orthonormal with respect to the standard inner product. This necessitates a careful definition of adjoint operators to ensure geometric consistency during map refinement.

#### 3.3.1. Functional Maps Under Orthogonal and Non-Orthogonal Bases

First, we clarify key symbols for consistency: Let F(S) denote the space of real-valued functions on shape *S*; arrange a general basis of its *k*-dimensional subspace into a matrix $\Phi_{k}=\left[\phi_{1},\ldots ,\phi_{k}\right]\in R^{n\times k}$. For non-orthogonal bases, the mass matrix in the spectral domain, defined as $M_{k}=\Phi_{k}^{T}M\Phi_{k}$, is not an identity matrix.

A fundamental step for restricted bases is computing the best approximation of a function f∈F(S) in $\langle \Phi_{k}\rangle$, which is defined by the orthogonal projection operator:

(4) $$\Phi_{k}^{†}f=\arg\min_{x\in R^{k}}{∥\Phi_{k}x-f∥}_{M}^{2}$$

where $\Phi_{k}^{†}$ denotes the pseudoinverse of $\Phi_{k}$ and ${∥\cdot ∥}_{M}$ is the mass-weighted norm.

In the standard functional map framework, the adjoint of the map matrix coincides with its transpose. However, due to the non-orthogonality of the elastic basis, the adjoint operator must be corrected by the mass matrices. The relationship between the functional map $C_{12}$ and the point-to-point map $P_{12}$ is given by

(5) $$C_{12}^{*}=\Phi_{2,k}^{†}P_{12}^{*}\Phi_{1,k}$$

This equation ensures that the geometric structure of the map is preserved in the spectral domain. We provide the detailed mathematical derivation of this relationship and the definition of the adjoint operators in Appendix A.

#### 3.3.2. Point-to-Point Map Calculation

To recover a precise point-to-point correspondence from the functional map, we enforce consistency between the spectral and spatial representations. The term $∥C_{12}C_{12}^{*}-I∥$ exhibits regularization properties, capturing shape differences and enhancing invertibility. Incorporating this into the objective function yields

(6) $$J\left(C_{12},P_{12}\right)={∥\Phi_{1,k}P_{12}\Phi_{2,k}C_{12}^{*}-\Phi_{1,k}∥}^{2}$$

where the norm ∥·∥ is defined on the orthogonal complement space of $\Phi_{1,k}$, imposing more comprehensive constraints on the continuity and consistency of $P_{12}$.

Minimizing *J* effectively aligns the spectral embeddings of the two shapes. The optimization with respect to the point-to-point map $\Pi_{12}=P_{12}^{T}$ is row-separable. For all j=1,…,n, the minimizer satisfies $\Pi_{12}\left(i,j\right)=1$ if and only if

(7) $$i=\arg\min_{r\in \{1,\ldots ,m\}}{∥M_{1,k}^{-1}{\left(\Phi_{2,k}C_{12}^{*}\right)}^{T}e_{r}-\Phi_{1,k}^{T}e_{j}∥}_{M_{1,k}}^{2}$$

This step can be interpreted as a nearest neighbor search in the aligned spectral embedding space (further details on the derivation are provided in Appendix B). We employ an alternating optimization framework (similar to ZoomOut [15]) to iteratively refine $C_{12}$ and $P_{12}$ by increasing the spectral resolution *k*.

### 3.4. Shape Distance Measurement

In machine learning, shape features are typically represented as high-dimensional vectors. To quantify the similarity between two shape feature vectors, cosine similarity [27] is employed—this metric focuses on the angular relationship between vectors, avoiding distortion from scalar differences. For two shapes quantized as *n*-dimensional feature vectors *A* and *B*, their cosine similarity is defined as follows:

(8) $$\cos\left(\theta \right)=\frac{A\times B}{∥A∥\times ∥B∥}$$

where A×B denotes the dot product of *A* and *B*, and ∥A∥,∥B∥ are the Euclidean (L2) norms of *A* and *B*, respectively.

Derived from cosine similarity, cosine distance quantifies the angular divergence between two vectors, making it particularly suitable for high-dimensional shape feature spaces. Its definition is

(9) CosineDistance=1−cos(θ)

The cosine distance ranges from 0 to 2: a value of 0 indicates identical vector directions (high shape similarity), while 2 indicates opposite directions (low similarity). Critically, this metric depends only on vector direction (not magnitude)—even if two shapes have structural or scalar differences, their cosine distance remains small if their feature vectors are directionally aligned. This property makes it ideal for normalized shape data, as it mitigates interference from non-isometric deformations (e.g., stretching and compression) and accurately captures the intrinsic similarity between geometric forms.

## 4. Experiments

This section details the experimental setup and results. We first introduce the datasets employed in our evaluation, then validate the effectiveness of the proposed elastic functional maps, and finally present the results for shape distance calculation and classification.

### 4.1. Experimental Datasets

To comprehensively evaluate the proposed framework, we selected three datasets with diverse characteristics, ranging from articulated objects to fine-grained anatomical surfaces. The SHREC 2015 dataset [28] and the McGill dataset [29] serve as public benchmarks, while a private Face dataset is introduced for high-fidelity deformation analysis.

SHREC 2015 dataset: The SHREC 2015 dataset comprises 1200 watertight 3D triangle meshes across 50 categories. It is a balanced dataset, where each category contains exactly 24 objects exhibiting distinct poses and topological variations. Designed primarily for non-rigid 3D shape retrieval, this dataset evaluates algorithmic robustness against shape deformation and topological changes.

McGill dataset: The McGill dataset consists of 255 objects with articulated parts, spanning 10 categories (e.g., ants, crabs, hands, and humans). This dataset is characterized by significant non-isometric articulated deformations, such as limb rotations and bending, which effectively tests the robustness of the algorithm against part-based variations.

Face dataset: This dataset contains 100 high-resolution 3D face mesh models, preserving detailed anatomical features. Collected specifically for this study, it is characterized by fine-grained geometric details and captures subtle non-isometric deformations caused by varying facial expressions. This high-fidelity data is particularly suitable for analyzing subtle surface variations.

Data Pre-processing: To ensure consistent input for spectral analysis, all 3D meshes underwent a standardized pre-processing pipeline. Initially, center-of-mass alignment was performed to normalize spatial positioning. Subsequently, to address scale ambiguity between models, we normalized the surface area of each mesh to unity. Finally, to establish the functional representation space, we constructed spectral basis functions on the normalized meshes, providing the essential geometric encoding for the subsequent SRE-FMaps framework.

### 4.2. Experimental Design

Parameter evaluation was conducted on the McGill dataset. We selected a representative pair of non-isometrically deformed human shapes and performed an ablation study to validate the effectiveness of the core components within our framework. Specifically, we compared multiple initialization strategies (nearest neighbor [NN] vs. Sinkhorn) and spectral bases (Laplace–Beltrami [LB] vs. elastic basis [EB]) by varying the number of basis functions. Performance was assessed in terms of both accuracy and efficiency using six widely adopted metrics: average geodesic error, bijectivity error, coverage, Chamfer distance, smoothness, and runtime.

For notational consistency, let $d_{S}\left(\cdot ,\cdot \right)$ and $d_{T}\left(\cdot ,\cdot \right)$ denote the geodesic distance on the source shape *S* and target shape *T*, respectively; Π the estimated correspondence; *A* and *B* two point sets of sizes N=|A| and M=|B|; and I(·) the indicator function. To quantitatively assess correspondence quality, we report six commonly used metrics.

Geodesic Error (GtErr, mm). This metric evaluates the intrinsic distortion induced by the mapping. Given a set of point pairs $\left(p_{i},q_{i}\right)$ on the source shape and their mapped counterparts on the target via correspondence Π, it is defined as

(10) $$\mathrm{GtErr}=\frac{1}{N}\sum_{i=1}^{N}|\;d_{S}\left(p_{i},q_{i}\right)-d_{T}\;\left(\Pi \left(p_{i}\right),\Pi \left(q_{i}\right)\right)\;|$$

Lower values indicate better preservation of intrinsic surface structure.

Bijectivity Error (%). To measure the consistency between forward and backward mappings, we compute

(11) $$\mathrm{BijErr}=\frac{1}{N}\sum_{i=1}^{N}I\;\left(\Pi_{YX}\;\left(\Pi_{XY}\left(i\right)\right)\neq i\right)\times 100\%$$

A perfectly bijective correspondence yields zero error.

Coverage (%). We further report the proportion of source points that are correctly mapped to valid targets:

(12) $$\mathrm{Cov}=\frac{N_{\mathrm{correct}}}{N}\times 100\%$$

Higher coverage reflects robustness against degeneration or collapse.

Chamfer Distance (mm^2^). To evaluate geometric proximity in Euclidean space, we compute the symmetric Chamfer distance

(13) $$\mathrm{CD}\left(A,B\right)=\frac{1}{N}\sum_{a\in A}\min_{b\in B}{∥a-b∥}^{2}+\frac{1}{M}\sum_{b\in B}\min_{a\in A}{∥b-a∥}^{2}$$

This complements GtErr by assessing visual and spatial alignment.

Smoothness (Dirichlet Energy, a.u.). The local regularity of the mapping function *f* is measured by

(14) $$E_{\mathrm{Dir}}\left(f\right)=\frac{1}{2}\int_{\Omega }{∥\nabla f∥}^{2}\;d\Omega$$

Lower values indicate smoother and more stable correspondences.

Runtime (s). We report the empirical wall-clock time *T* and additionally list the theoretical complexity

(15) $$T\;\left(s\right),\;C\;=\;O\;\left(n_{0}\times N\times k_{0}\right)$$

This presentation provides insight into both the practical efficiency and scalability of the proposed method.

**Figure 3:**
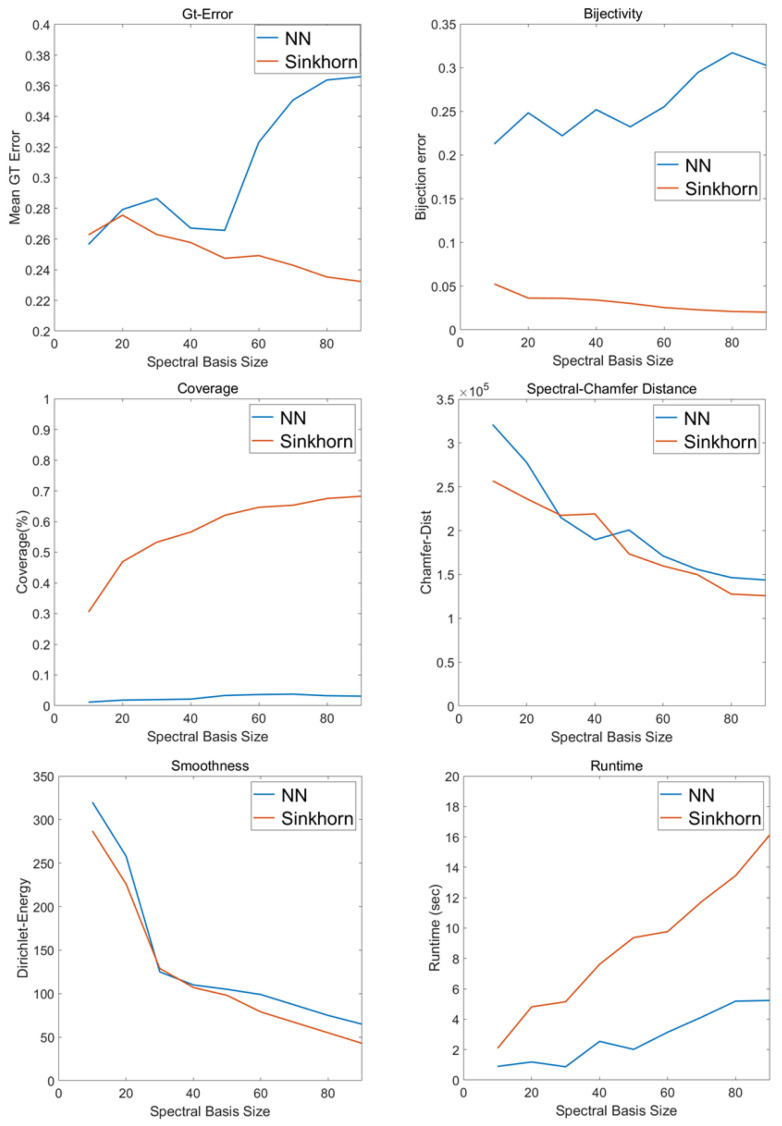
Comparison of NN and Sinkhorn under different basis numbers.

As shown in Figure 3, the six evaluation metrics of the nearest neighbor (NN) and Sinkhorn initialization strategies were compared under varying numbers of basis functions. For geodesic error, NN exhibits a decreasing–increasing trend, whereas Sinkhorn consistently maintains lower distortion and achieves its minimum at 90 bases. For bijectivity error, NN fluctuates strongly, while Sinkhorn remains stable and steadily improves as the basis size increases. Coverage further highlights the advantage of Sinkhorn: NN yields almost no valid matches (close to 0%), whereas Sinkhorn improves monotonically and stabilizes at approximately 70% when 90 bases are used. The Chamfer distance of both methods decreases rapidly at first and then gradually levels off, with Sinkhorn achieving the lowest value at 90 bases. Regarding smoothness, the Dirichlet energy of both mappings decreases with increasing basis size, yet Sinkhorn consistently yields smoother functional assignments. Finally, the runtime of both methods increases with the number of bases, and Sinkhorn incurs a higher computational cost than NN.

Synthesizing these metric evaluations, we further analyze the optimal parameter selection and the algorithm’s scalability to balance accuracy and efficiency.

First, regarding the parameter selection, the number of basis functions (*k*) represents a critical trade-off between spectral resolution and computational noise. As analyzed in Figure 3, setting k<50 results in the loss of high-frequency geometric details, while k>100 introduces spectral instability and significantly increases the complexity of Sinkhorn iterations. Consequently, based on the convergence of error metrics, k=90 was empirically selected as the optimal balance.

Second, addressing the computational cost noted above, we analyze the scalability of the framework. The complexity of SRE-FMaps is primarily dominated by the Sinkhorn initialization ($O(N^{2})$). While this is efficient for standard datasets (e.g., ∼5–10 k vertices), the runtime scales quadratically with mesh resolution. To mitigate this for very large meshes (>50 k vertices), we recommend utilizing the proposed sparse kernel approximation, which reduces the effective complexity to near-linear (O(NlogN)), ensuring practical applicability in industrial scenarios.

#### Matching Performance Evaluation

**Figure 4:**
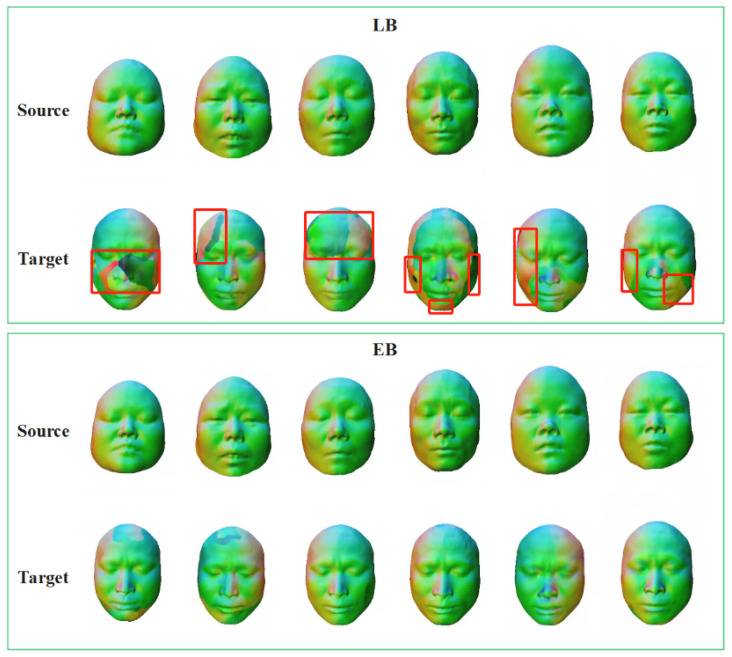
Comparison of the visualization results of the LB operator and elastic basis. Red boxes indicate the regions with inconsistent correspondences.

After obtaining the initial correspondence, we further validate the effectiveness of elastic functional maps by comparing the Laplace–Beltrami (LB) operator with the elastic basis (EB) on the Face dataset. As shown in Figure 4, LB aligns prominent regions such as the nose and mouth but exhibits noticeable mismatches in flatter or highly deformable areas (e.g., cheeks and chin), resulting in fragmented or distorted color transfer. In contrast, EB produces globally consistent correspondences with smooth region transitions, indicating higher robustness and stability under non-isometric variations.

**Figure 5:**
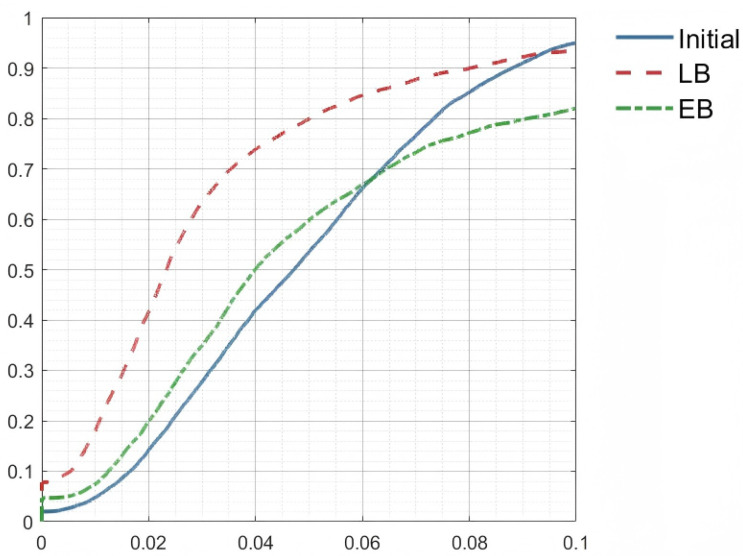
Visual correspondence comparison between the Laplace–Beltrami (LB) operator and the elastic basis (EB) on the Face dataset.

A quantitative comparison on the McGill human pair further supports this observation. Figure 5 reports the cumulative correspondence ratio under varying point-to-point error thresholds for Initial, LB, and EB mappings. EB rises the fastest in the low-error range and consistently remains the highest across all thresholds, demonstrating the smallest alignment error and the largest proportion of reliable matches. LB performs moderately, while the Initial map retains the fewest valid correspondences. Overall, EB exhibits superior accuracy and error tolerance, confirming its advantage in non-isometric shape matching.

### 4.3. Comparative Experiment

**Figure 6:**
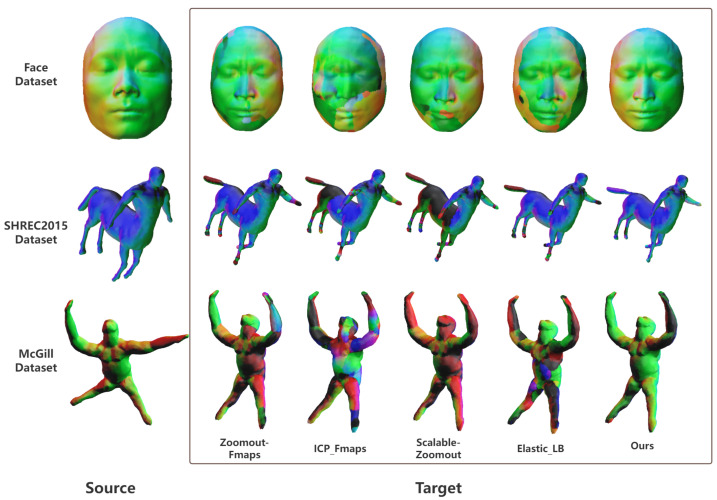
Comparison between ZoomOut, ICP, Scalable-ZoomOut, EB, and our method on three representative datasets (Face, SHREC2015, and McGill).

To validate the effectiveness of our approach, we compare it against representative functional map-based methods on three datasets (Face, SHREC2015, and McGill), with qualitative correspondence visualizations shown in Figure 6. Across all datasets, our method achieves markedly better structural continuity and alignment accuracy.

On the Face dataset, classical methods (ZoomOut, ICP, and Scalable-ZoomOut) exhibit color discontinuities in fine-scale regions such as the nasal bridge and mouth corners, while the elastic basis (EB) variant smooths global transitions but blurs local features. In contrast, our method preserves both natural gradients and precise feature boundaries. A similar pattern is observed on SHREC2015 (centaur shapes) and McGill (gorilla shapes), where competing methods introduce deformation artifacts at limb joints or extremities, whereas our approach maintains coherent and geometrically consistent correspondences.

It is important to note that our comparisons are restricted to methods within the functional map (FM) framework, as the proposed elastic functional maps specifically target limitations of classical FM pipelines—such as reliance on isometric assumptions and post-processing refinements. Direct comparison with neural models (e.g., FMNet [7] and Deep Shells [19]) would introduce additional learning-related variables and obscure the contribution of our geometric regularization strategy.

Overall, the visual evidence confirms that our method produces more accurate and continuous correspondences than existing FM-based approaches, demonstrating greater robustness under non-isometric deformation.

### 4.4. Shape Distance Calculation and Classification Results

#### 4.4.1. Shape Distance Calculation

For the Face dataset, six shapes were used and divided into two classes (three male and three female). For the SHREC2015 dataset, six representative categories (centaur, ant, gorilla, pliers, dog, and human) were selected, with one shape per class. Table 1 summarizes the cosine distance results of four correspondence strategies (NN + LB, NN + EB, Sinkhorn + LB, and Sinkhorn + EB). On the Face dataset, a smaller intra-class cosine distance indicates stronger similarity among samples of the same gender, while a larger inter-class distance reflects better discriminative ability. Among all compared methods, the Sinkhorn+EB configuration achieves the lowest intra-class distances while maintaining clear inter-class separation, suggesting improved feature compactness and better class separability.

#### 4.4.2. Classification Results

**Figure 7:**
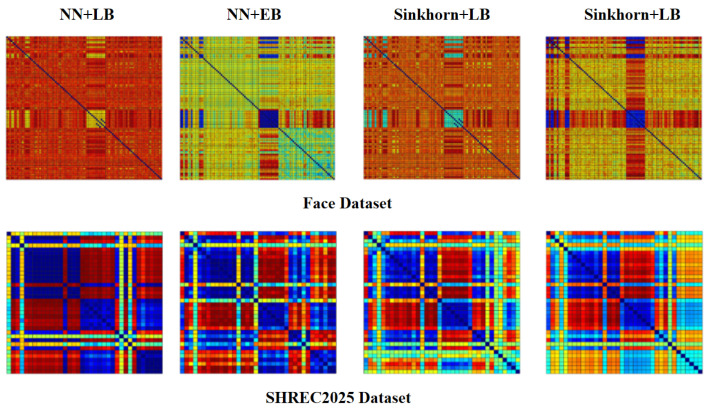
Cosine distance heatmaps generated by different correspondence strategies. In the heatmaps, warmer colors (e.g., red, yellow) represent smaller cosine distances (higher similarity), while cooler colors (e.g., blue, cyan) represent larger cosine distances (lower similarity). Sinkhorn + EB exhibits clear block-diagonal structures with high intra-class compactness and inter-class separability on both Face and SHREC2015 datasets.

Figure 7 illustrates the cosine distance heatmaps for both datasets. On the Face dataset, the Sinkhorn + EB configuration exhibits clear block-diagonal structures, characterized by compact intra-class regions and sharply defined inter-class boundaries. A similar trend is observed on SHREC2015, where six categories are cleanly separated without noticeable cross-category leakage. In contrast, the remaining methods suffer from either intra-class dispersion or inter-class entanglement. These results suggest that Sinkhorn + EB produces more stable and discriminative correspondence patterns in both binary and multi-class settings.

To further validate classification robustness under different task complexities, two representative datasets were employed. The Face dataset corresponds to a binary scenario, where 99 models were split into 80% training and 20% testing sets. The SHREC2015 dataset represents a more challenging six-class setting, with a 70%/30% train–test split over 35 models.

Performance was evaluated using standard global metrics (accuracy, precision, recall, and F1-score), complemented by specificity, which is particularly informative for negative-sample detection in binary classification (see Table 2). The mathematical definitions of these standard performance metrics are adopted from [30]. Across all correspondence strategies (NN + LB, NN + EB, Sinkhorn + LB, and Sinkhorn + EB), the proposed Sinkhorn+EB consistently achieves superior results on both datasets (see Table 3 and Table 4). On the Face dataset, it achieves 94.74% accuracy with perfect specificity and precision (100%), while retaining an 88.89% recall rate (F1 = 0.94), indicating that it can detect positive samples effectively without introducing false alarms. On SHREC2015, despite higher inter-class diversity, Sinkhorn+EB still maintains balanced precision and recall at 91.67% (F1 = 0.89), outperforming all baselines.

Taken together, these results demonstrate the complementary benefits of Sinkhorn optimization and elastic basis construction. While Sinkhorn improves correspondence stability over nearest neighbor initialization, the elastic basis contributes additional discriminative power by capturing extrinsic deformation cues. Their combination yields both intra-class compactness and inter-class separability, whereas NN+LB notably suffers from severe recall degradation (only 33.33% on Face), revealing its limitations in handling complex deformations.

### 4.5. Discussion and Limitations

This study presented SRE-FMaps, a framework designed to address the limitations of Laplace–Beltrami bases in handling non-isometric deformations. The primary outcome of our study, as evidenced by the experiments on the Face, McGill, and SHREC2015 datasets, is that replacing the standard spectral basis with a Hessian-derived elastic basis significantly enhances the preservation of high-frequency features under large deformations. Furthermore, the integration of entropy-regularized Sinkhorn transport provides a globally consistent initialization that avoids the local minima often encountered by nearest neighbor approaches.

To validate the individual contributions of these components, we conducted an ablation study by comparing four configurations: NN + LB, NN + EB, Sinkhorn + LB, and Sinkhorn + EB (Table 1, Table 3, and Table 4). The quantitative results demonstrate that while the elastic basis (EB) alone improves local detail preservation, its combination with Sinkhorn initialization yields the highest accuracy and robustness. This confirms that a bijective and globally consistent initialization is a prerequisite for fully leveraging the deformation-aware properties of the elastic basis. Specifically, the elastic basis (EB) encodes the local minimization of the elastic shell energy, making the basis functions inherently robust to non-isometric changes like local bending and folding. This mechanism allows SRE-FMaps to naturally preserve high-curvature details during functional map matching, a capability often degraded by standard spectral methods.

Although the proposed SRE-FMaps framework demonstrates strong robustness, several limitations remain. First, when encountering extreme distortions (e.g., stretching or compression exceeding 50%), the high-frequency components of the elastic basis become less stable, leading to a noticeable degradation in correspondence precision due to weakened locality of the elastic shell eigenfunctions.

Second, a limitation lies in the construction of the sparse kernel matrix, where the neighborhood size N(i) must be manually specified. While a fixed value performs well within a dataset, the optimal setting varies across shape categories and deformation types, indicating the lack of an adaptive selection mechanism. Furthermore, the iterative refinement still requires multiple steps, which may become computationally expensive on very large point sets.

Future work will address these limitations through two dimensions. To overcome the manual parameter selection, we plan to implement an adaptive neighborhood strategy based on local curvature entropy, allowing N(i) to vary dynamically according to geometric complexity. Additionally, to handle extreme topological variations and improve computational efficiency, we will explore a coarse-to-fine multi-scale optimization scheme that progressively refines the elastic basis, ensuring stability even under severe non-isometric distortions, and investigate multi-resolution solvers for large-scale dense point clouds.

## 5. Conclusions

This paper presents an elastic functional map-based algorithm for non-isometric 3D shape matching. To address the instability of traditional Laplace–Beltrami eigenbases and the misalignment caused by large deformations, correspondence is initialized using the Sinkhorn algorithm, yielding bijective and globally consistent mappings via a sparse entropy-regularized kernel. A non-orthogonal elastic basis derived from the Hessian of elastic shell energy further enhances local sensitivity and high-frequency responsiveness. Extensive experiments on the Face, McGill, and SHREC2015 datasets confirm that the proposed framework outperforms existing approaches in terms of matching accuracy, robustness, and classification capability, particularly under high-curvature bending and localized folding. These results demonstrate that SRE-FMaps offers a practical and efficient solution for real-world non-isometric shape correspondence. However, limitations remain regarding the stability of basis functions under extreme distortions (e.g., excessive stretching) and the reliance on manual parameter tuning for kernel construction, which may incur high computational costs on large-scale dense point clouds. To overcome these limitations, future work will focus on exploring curvature-aware or entropy-driven strategies to automatically adjust the neighborhood size N(i), as well as investigating multi-resolution solvers and data-driven refinement of elastic bases to further enhance scalability and robustness against extreme topological variations. 

## Figures and Tables

**Table 1:** Cross-dataset method performance comparison. For the Face dataset, C1 = Male and C2 = Female. For the SHREC2015 dataset, C1 = Centaur, C2 = Ant, C3 = Gorilla, C4 = Pliers, C5 = Dog, and C6 = Human. Repeated combinations (e.g., C1-C1) indicate different subject pairs from the same class.

Dataset	Method Combinations				
	Combination	NN + LB	NN + EB	Sink + LB	Sink + EB
Face	Same Kinds				
	C1-C1	0.7855	0.5009	0.4287	0.1102
	C1-C1	0.7968	0.6804	0.5235	0.3222
	C1-C1	0.8737	0.7487	0.6090	0.1385
	C2-C2	0.7966	0.5881	0.5354	0.1511
	C2-C2	0.8282	0.6203	0.5912	0.3108
	C2-C2	0.8141	0.6958	0.4683	0.2625
	Different Kinds				
	C1-C2	1.0900	1.2306	1.2268	1.3661
	C1-C2	1.1048	1.3157	1.0267	1.2904
	C1-C2	1.0034	1.1074	0.9630	1.0196
SHREC2015	Same Kinds				
	C1-C1	0.8801	0.5398	0.6698	0.5139
	C2-C2	0.3557	0.4360	0.3239	0.2192
	C3-C3	0.3051	0.2918	0.3433	0.1009
	C4-C4	0.6438	0.5015	0.4841	0.1755
	C5-C5	0.6985	0.3761	0.4750	0.2750
	C6-C6	0.3099	0.3030	0.8203	0.6367
	Different Kinds				
	C1-C2	1.0295	1.1799	1.6532	1.7381
	C1-C3	1.3148	1.4316	1.6126	1.7893
	C1-C4	0.7150	0.9408	1.5563	1.5693
	C1-C5	1.3621	1.3885	1.3693	1.6932
	C1-C6	1.1759	1.7381	1.3306	1.3701

**Table 2:** Commonly used classification evaluation indicators.

Evaluation Indicators	Calculation Formula	Description
Accuracy	$\frac{TP+TN}{TP+FP+TN+FN}$	Accuracy is the most intuitive global performance indicator, measuring the proportion of correct predictions made by the model as a whole.
Precision	$\frac{TP}{TP+FP}$	Precision is the proportion of samples predicted to be positive that are actually positive, reflecting the reliability of the model’s prediction results.
Recall	$\frac{TP}{TP+FN}$	The recall rate is the proportion of positive samples that are correctly identified, reflecting the ability of the model to find positive samples.
Specificity	$\frac{TN}{TN+FP}$	The specificity is the proportion of correctly identified samples in the negative class, which reflects the ability of the model to identify negative samples and is only used in binary classification.
F1-Score	$2\times \frac{\mathrm{Precision}\times \mathrm{Recall}}{\mathrm{Precision}+\mathrm{Recall}}$	The F1-score is the harmonic average of precision and recall, which comprehensively evaluates the accuracy and coverage of the model.

**Table 3:** Comparison of classification performance on the Face dataset.

Metric	Methods			
	NN + LB	NN + EB	Sinkhorn + LB	Sinkhorn + EB
Accuracy (%)	42.11	63.16	73.68	94.74
Precision (%)	57.14	63.64	77.78	100.00
Recall (%)	33.33	70.00	70.00	88.89
Specificity (%)	57.14	55.56	77.78	100.00
F1-Score	0.42	0.67	0.74	0.94

**Table 4:** Comparison of classification performance on the SHREC2015 dataset.

Metric	Methods			
	NN + LB	NN + EB	Sinkhorn + LB	Sinkhorn + EB
Accuracy (%)	40.00	60.00	70.00	90.00
Precision (%)	33.33	52.78	66.67	91.67
Recall (%)	33.33	58.33	61.11	91.67
F1-Score	0.31	0.55	0.61	0.89

## Data Availability

The original data presented in the study are openly available in SHREC 2015 dataset at https://www.icst.pku.edu.cn/zlian/representa/3d15/dataset/index.htm (accessed on 15 October 2025) and in the McGill 3D Shape Benchmark at http://www.cim.mcgill.ca/~shape/benchMark/ (accessed on 15 October 2025), but currently unavailable due to webpage parsing failure. Alternative access can be requested from the McGill University School of Computer Science or via academic data repositories such as IEEE DataPort).

## Appendix A. Derivation of Adjoint Operators

In this appendix, we provide the detailed mathematical definitions for the inner products and derive the adjoint operators referenced in Section 3.2 and Section 3.3. We first define the general continuous case and then derive the specific matrix forms required for non-orthogonal elastic bases.

### Appendix A.1. Continuous Definitions and Inner Products

Let F(X) and F(Y) denote the spaces of real-valued functions on source shape *X* and target shape *Y*, respectively. The standard $L^{2}$ inner product of two functions f,g∈F(X) is defined as follows:

(A1) $${\langle f,g\rangle }_{X}=\int_{X}f\left(x\right)g\left(x\right)\;dx$$

In the context of functional maps, a linear operator $T_{yx}^{F}:F\left(X\right)\to F\left(Y\right)$ transfers functions from *X* to *Y*. The adjoint operator $T_{xy}^{A}:F\left(Y\right)\to F\left(X\right)$ is implicitly defined by the following adjoint property, which must hold for all f∈F(X) and g∈F(Y):

(A2) $${\langle T_{xy}^{A}\left(g\right),f\rangle }_{X}={\langle g,T_{yx}^{F}\left(f\right)\rangle }_{Y}$$

This property forms the theoretical basis for converting functional correspondence back to spatial point-to-point correspondence.

### Appendix A.2. Discrete Discretization and Mass Matrices

When shapes are discretized into meshes with $n_{1}$ and $n_{2}$ vertices, functions are represented as vectors. Let $M_{1}\in R^{n_{1}\times n_{1}}$ and $M_{2}\in R^{n_{2}\times n_{2}}$ be the diagonal lumped mass matrices (containing area weights) for shapes $S_{1}$ and $S_{2}$. The discrete inner product becomes

(A3) $${\langle f,g\rangle }_{M_{i}}=f^{T}M_{i}g$$

For the specific case of SRE-FMaps, we employ a truncated elastic basis. Let $\Phi_{i,k}\in R^{n_{i}\times k}$ be the matrix containing the first *k* elastic basis functions. The spectral mass matrix in this reduced basis is defined as follows:

(A4) $$M_{i,k}=\Phi_{i,k}^{T}M_{i}\Phi_{i,k}\in R^{k\times k}$$

Note: Unlike the Laplace–Beltrami eigenbasis, the elastic basis is generally non-orthogonal, meaning $M_{i,k}\neq I$. This requires explicit correction terms in the adjoint derivations below.

### Appendix A.3. Adjoint of the Functional Map Matrix

Let $C_{12}\in R^{k\times k}$ be the functional map matrix (the discrete representation of $T^{F}$) transferring coefficients from basis $\Phi_{1,k}$ to $\Phi_{2,k}$. Its adjoint matrix $C_{12}^{*}$ (representing $T^{A}$) is derived from the discrete version of Equation (A2):

(A5) $${\langle C_{12}a,b\rangle }_{M_{2,k}}={\langle a,C_{12}^{*}b\rangle }_{M_{1,k}}$$

where $a,b\in R^{k}$ are coefficient vectors. This can be expanded using the inner product definition:

(A6) $$\begin{array}{cc} {\left(C_{12}a\right)}^{T}M_{2,k}b & =a^{T}M_{1,k}\left(C_{12}^{*}b\right) \end{array}$$

(A7) $$\begin{array}{cc} a^{T}C_{12}^{T}M_{2,k}b & =a^{T}M_{1,k}C_{12}^{*}b \end{array}$$

Since this holds for arbitrary vectors, we obtain the closed-form expression for the adjoint functional map:

(A8) $$C_{12}^{*}=M_{1,k}^{-1}C_{12}^{T}M_{2,k}$$

### Appendix A.4. Adjoint of the Point-to-Point Map

Similarly, let $P_{12}\in R^{n_{2}\times n_{1}}$ be the binary matrix representing the point-to-point correspondence. Its adjoint $P_{12}^{*}$ satisfies ${\langle P_{12}f,g\rangle }_{M_{2}}={\langle f,P_{12}^{*}g\rangle }_{M_{1}}$. Following the same derivation logic as above yields

(A9) $$P_{12}^{*}=M_{1}^{-1}P_{12}^{T}M_{2}$$

## Appendix B. Derivation of Spectral–Spatial Consistency

In this appendix, we utilize the adjoint operators derived in Appendix A to establish the relationship between the functional map and the point map in the elastic basis. This leads to the consistency equation used in Equation (5) of the main text.

The functional map $C_{12}$ is computed by projecting the mapped basis functions. Using the generalized pseudoinverse $\Phi_{i,k}^{†}=M_{i,k}^{-1}\Phi_{i,k}^{T}M_{i}$, we have:

(A10) $$C_{12}=\Phi_{2,k}^{†}P_{12}\Phi_{1,k}$$

To find the adjoint $C_{12}^{*}$, we substitute this into Equation (A8) from Appendix A:

(A11) $$C_{12}^{*}=M_{1,k}^{-1}{\left(\Phi_{2,k}^{†}P_{12}\Phi_{1,k}\right)}^{T}M_{2,k}$$

Expanding the transpose (noting that ${\left(\Phi^{†}\right)}^{T}=M\Phi M^{-1}$ due to mass symmetry),

(A12) $$C_{12}^{*}=M_{1,k}^{-1}\Phi_{1,k}^{T}P_{12}^{T}\left(M_{2}\Phi_{2,k}M_{2,k}^{-1}\right)M_{2,k}$$

The term $M_{2,k}^{-1}M_{2,k}$ cancels to identity:

(A13) $$C_{12}^{*}=M_{1,k}^{-1}\Phi_{1,k}^{T}P_{12}^{T}M_{2}\Phi_{2,k}$$

By inserting $M_{1}M_{1}^{-1}$ appropriately, we recover the adjoint definitions:

(A14) $$C_{12}^{*}=\underset{\Phi_{1,k}^{†}}{\underset{︸}{\left(M_{1,k}^{-1}\Phi_{1,k}^{T}M_{1}\right)}}\underset{P_{12}^{*}}{\underset{︸}{\left(M_{1}^{-1}P_{12}^{T}M_{2}\right)}}\Phi_{2,k}$$

Thus, we arrive at the consistency equation used in the main text:

(A15) $$C_{12}^{*}=\Phi_{1,k}^{†}P_{12}^{*}\Phi_{2,k}$$

This confirms that our framework preserves the geometric structure of the map even when using non-orthogonal elastic bases.
